# Comparative Biological and Functional Profiling of Single-Position Cysteine Substitutions in the HNP-1-Derived Peptide Pep-H Against *Mycobacterium tuberculosis*

**DOI:** 10.3390/antibiotics15060618

**Published:** 2026-06-17

**Authors:** Christian S. Carnero Canales, Letícia Oliveira Catarin Nunes, Ariani Rodrigues Aragão, Norival Alves Santos Filho, Roxana Yesenia Pastrana Alta, Fernando Rogério Pavan

**Affiliations:** 1 BIOMET, Laboratorio de Química Bioinorgánica en Medicina, Medioambiente y Tecnología, Facultad de Ciencias, Universidad Nacional de Ingeniería, Av. Túpac Amaru 210, Rímac, Lima 15333, Peru; rpastranaa@uni.edu.pe; 2 School of Pharmaceutical Sciences, São Paulo State University (UNESP), Araraquara 14800-903, Brazil; leticia.catarin@unesp.br (L.O.C.N.); norival.santos-filho@unesp.br (N.A.S.F.); 3 Institute of Chemistry, São Paulo State University, Araraquara 14800-060, Brazil; ariani.rodrigues@unesp.br

**Keywords:** antimicrobial peptides, Pep-H, cysteine, structure–activity relationship, *Mycobacterium tuberculosis*, HNP-1-derived peptides, efflux pumps, antioxidant activity

## Abstract

**Background/Objectives:** Tuberculosis remains a major public health challenge due to the persistence of *Mycobacterium tuberculosis* (*Mtb*) and the emergence of multidrug-resistant strains. In this study, Pep-H, an HNP-1-derived antimicrobial peptide with the sequence RRYGTCIYQGRLWAF-NH_2_, was used as a compact scaffold to examine how single-residue substitutions at the Cys position affected its biological and functional profile. **Methods:** A focused single-position substitution panel was generated by replacing Cys with Trp, Ala, Arg, or Met while preserving peptide length and sequence context, and the analogs were computationally prioritized according to their predicted antitubercular potential and contrasting side-chain properties. The peptides were synthesized, purified, characterized by HPLC and mass spectrometry, and evaluated for activity against *Mtb* H37Rv, cytotoxicity, hemolysis, ethidium bromide accumulation, and DPPH radical scavenging. **Results**: Pep-H retained the most favorable profile, showing the highest antimycobacterial potency, low hemolysis, favorable selectivity indices, enhanced ethidium bromide accumulation, and the strongest antioxidant response. All Cys substitutions reduced antimycobacterial activity, indicating that none of the tested residues reproduced the integrated biological profile of Pep-H. **Conclusions:** The contrasting outcomes of the Arg- and Met-containing analogs suggest that increased cationicity or sulfur retention alone was insufficient, while supporting a multifactorial contribution of Cys side-chain chemistry and the local GTCIY environment.

## 1. Introduction

The global persistence of tuberculosis is closely associated with the ability of *Mycobacterium tuberculosis* (*Mtb*) to develop multidrug resistance and survive under complex physiological conditions within the host, which continues to limit therapeutic efficacy and complicate the clinical and epidemiological control of the disease [1,2]. The pathogenic success of *Mtb* is associated with physiological and metabolic traits that promote persistence, immune evasion, host adaptation, and progression throughout the infection–disease cycle [3]. Part of this resilience depends on its cell envelope, organized into a mycolyl–arabinogalactan–peptidoglycan complex and a lipid-rich mycomembrane that acts as a permeability barrier and contributes to limiting the entry of numerous antimicrobial compounds [4].

Antimicrobial peptides (AMPs) have attracted attention as therapeutic candidates because of their structural diversity and their ability to act through complementary mechanisms, including membrane disruption, interaction with intracellular targets, and modulation of the immune response [5]. Beyond direct microbicidal activity, several AMPs and host defense peptides can also participate in antibiofilm, immunomodulatory, and infection-controlling processes, expanding their interest as anti-infective platforms [6]. The biological performance of AMPs depends on the balance between their physicochemical properties and structural organization. Therefore, rational design seeks to adjust peptide sequences to improve antimicrobial activity and stability, considering that even single substitutions may significantly alter hydrophobicity, potency, or cytotoxicity [7]. In the case of *Mtb*, these properties cannot be directly extrapolated from conventional antibacterial models, making experimental validation in mycobacterial systems necessary.

Human neutrophil peptide-1 (HNP-1) belongs to the defensin family of cationic host defense peptides produced by neutrophils and epithelial-related compartments that contribute to innate immunity through antimicrobial and immunoregulatory mechanisms [8]. A short HNP-1-derived motif, RRYGTCIYQGRLWAF-NH_2_, previously named Pep-H, was identified in silico and experimentally evaluated for antimycobacterial activity against *Mtb* in both free and nanoparticle-delivered forms [9]. Within this motif, cysteine represents a chemically distinctive residue because its thiol group provides tunable acidity, high nucleophilicity, access to multiple oxidation states, and participation in thiol-dependent redox buffering mechanisms [10].

In this study, Pep-H was used as a compact parental scaffold to investigate the chemical and positional contribution of Cys within the RRYGTCIYQGRLWAF-NH_2_ motif. A focused single-position substitution panel was generated by replacing Cys with Trp, Ala, Arg, or Met, thereby introducing contrasting but interdependent changes in side-chain chemistry and physicochemical properties. The analogs prioritized by the machine-learning models were subjected to a common experimental workflow comprising peptide synthesis and characterization, antimycobacterial activity against *Mtb* H37Rv, cytotoxicity, hemolysis, ethidium bromide accumulation, and antioxidant activity. Cytotoxicity was included to determine whether the selected substitutions affected mammalian-cell compatibility differently from antimycobacterial activity and to enable calculation of selectivity indices. This strategy enabled a chemically informed comparison of distinct residues at a fixed sequence position and provided an experimental basis for identifying substitution-dependent trends across antimycobacterial activity, host–cell compatibility, hemolysis, EtBr accumulation, and antioxidant behavior.

## 2. Results

### 2.1. Computational Design of a Single-Position Substitution Panel

A virtual library of 20 peptides was designed from the HNP-1-derived motif RRYGTCIYQGRLWAF-NH_2_, hereafter referred to as Pep-H, using a systematic single-residue substitution strategy in which the cysteine residue was individually replaced by each standard amino acid. All variants retained the same sequence length and overall sequence context, following the general format RRYGT**X**IYQGRLWAF, where X corresponds to the substituted residue. This approach enabled the evaluation of a single sequence position while maintaining the remaining peptide scaffold unchanged. The complete virtual library and the computational models employed are presented in Tables S1–S5.

The generated sequences were evaluated using AntiTBPred through four antitubercular prediction models: molecular dataset support vector machine (MD-SVM), molecular dataset hybrid (MD-hybrid), residue dataset support vector machine (RD-SVM), and residue dataset hybrid (RD-hybrid). AntiTBPred employs machine learning-based models to estimate antimycobacterial potential from sequence-derived features and amino acid composition, while its design module enables the generation and ranking of single-substitution mutants according to predictive scores.

From the virtual library, five AMPs were selected for synthesis and subsequent biological evaluation (Table 1). The selected analogs represented substitutions with contrasting physicochemical properties at the same sequence position. Tryptophan introduced an indole ring, increasing local hydrophobicity; alanine minimized the chemical contribution of the side chain; arginine increased cationic character through the incorporation of a guanidinium group; methionine preserved a sulfur atom in the form of a thioether; and cysteine retained the native thiol group.

All five peptides were classified as antitubercular by the four predictive models evaluated. The estimated physicochemical properties revealed a compact, cationic, and low-molecular-weight peptide series. CCC01, CCC02, Pep-H, and CCC05 displayed an estimated net charge of +3, whereas CCC03 reached +4 due to the incorporation of Arg. Molecular weights ranged approximately from 1858 to 1973 Da, maintaining all analogs within a range compatible with short synthetic peptides. The selected substitutions established a comparative chemical framework spanning cationicity, aromaticity, side-chain dimensions, and sulfur chemistry. Stability analysis using PlifePred predicted comparable half-lives among the five peptides, with estimated values ranging from 1015.91 to 1197.51 s. The predicted stability order was CCC02, CCC01, CCC05, Pep-H, and CCC03 (Table 2). The hemolytic prediction generated using HemoPI2 classified all five peptides as non-hemolytic. Evolutionary Scale Modeling scores ranged from 0.235 to 0.344, while the MERCI and hybrid models likewise classified all five peptides as non-hemolytic (Table S6).

### 2.2. Peptide Synthesis, Purification, and Characterization by Chromatography and Mass Spectrometry

**Figure 1:**
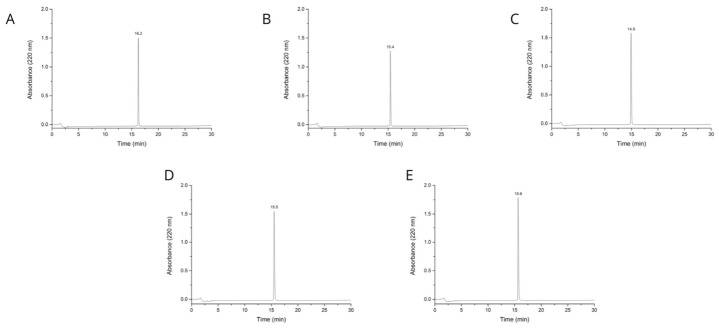
Chromatographic profile of the purified AMPs from the RRYGTXIYQGRLWAF series. Analytical chromatograms were recorded at 220 nm after chromatographic purification. All purified peptides showed chromatographic purity above 98%, as determined by analytical HPLC peak integration. (**A**) CCC01; (**B**) CCC02; (**C**) CCC03; (**D**) Pep-H; and (**E**) CCC05.

The five selected peptide analogs were successfully obtained and subjected to chromatographic purification (Figure 1). Initial chromatograms recorded at 220 nm showed a predominant signal for each synthesis product, accompanied in some cases by secondary peaks of lower intensity (Figure S1). After purification, analytical chromatograms displayed dominant peaks with defined retention times for each AMP, corresponding to 16.2 min for CCC01, 15.4 min for CCC02, 14.9 min for CCC03, 15.5 min for Pep-H, and 15.6 min for CCC05. The presence of a predominant chromatographic signal in the post-purification profiles supported the isolation of individual peptide products for subsequent biological evaluation of the series. Integration of the analytical HPLC chromatograms confirmed high chromatographic purity for all purified peptides, with purity values above 98% for CCC01, CCC02, CCC03, Pep-H, and CCC05.

Positive-mode mass spectrometry analysis revealed multicharged ion signals consistent with the expected molecular masses of the synthesized sequences (Figure S1). CCC01 displayed major signals at *m*/*z* 658.3541 and 494.0170, compatible with triply and quadruply charged species. CCC02 showed signals at *m*/*z* 620.0057 and 465.2558, whereas CCC03 exhibited signals at *m*/*z* 648.3609 and 486.5230. Pep-H presented signals at *m*/*z* 630.6624 and 472.9975, consistent with the sequence retaining Cys. CCC05 showed signals at *m*/*z* 640.0067 and 480.2565, corresponding to multicharged species of the Met-containing analog. These chromatographic and mass spectrometric data confirmed the successful purification and molecular identity of the five AMPs used for biological evaluation.

### 2.3. Antimycobacterial Activity Against Mtb H37Rv

The antimycobacterial activity of the AMPs was evaluated against *Mtb* H37Rv (Table 3). Considering that lower values indicate higher potency, Pep-H showed the best performance within the series, with a value of 24.37 ± 0.07 μg mL^−1^. The analogs generated by Cys substitution displayed higher values, corresponding to 45.25 ± 1.01 μg mL^−1^ for CCC03, 46.64 ± 0.54 μg mL^−1^ for CCC05, 61.61 ± 7.16 μg mL^−1^ for CCC01, and 94.61 ± 2.19 μg mL^−1^ for CCC02.

Direct comparison with Pep-H showed that substitution of Cys reduced antimycobacterial potency throughout the entire series. CCC03, which incorporates Arg at the evaluated position, displayed a value 1.86-fold higher than Pep-H. CCC05, in which Cys was replaced by Met, showed a 1.91-fold increase. CCC01, containing Trp, was 2.53-fold less potent than Pep-H, whereas CCC02, containing Ala, exhibited the greatest relative loss, with a value 3.88-fold higher than the Cys-containing peptide. Retention of Cys in Pep-H was associated with the highest antimycobacterial activity of the series. Substitution with Arg generated the analog most similar to Pep-H, followed by Met, whereas Trp and Ala produced a more pronounced reduction in potency.

Pep-H and CCC05 both contain sulfur-bearing residues at the evaluated position; however, only Pep-H retains the Cys thiol. The lower potency of CCC05 indicates that sulfur retention as a thioether was insufficient to preserve the activity of Pep-H. Because Met also introduces a longer side chain and places sulfur farther from the peptide backbone, this comparison supports a contribution of Cys-specific chemistry and local geometry rather than an exclusively electronic explanation.

Activity against Mtb H37Rv showed a clear dependence on the identity of residue X within the RRYGTXIYQGRLWAF motif. Within this sequence context, Arg and Met were better tolerated than Trp and Ala, but none of these substitutions reproduced the potency associated with Cys.

### 2.4. Cytotoxicity in Human Lung Fibroblasts MRC-5 and Murine Macrophages RAW 264.7

**Figure 2:**
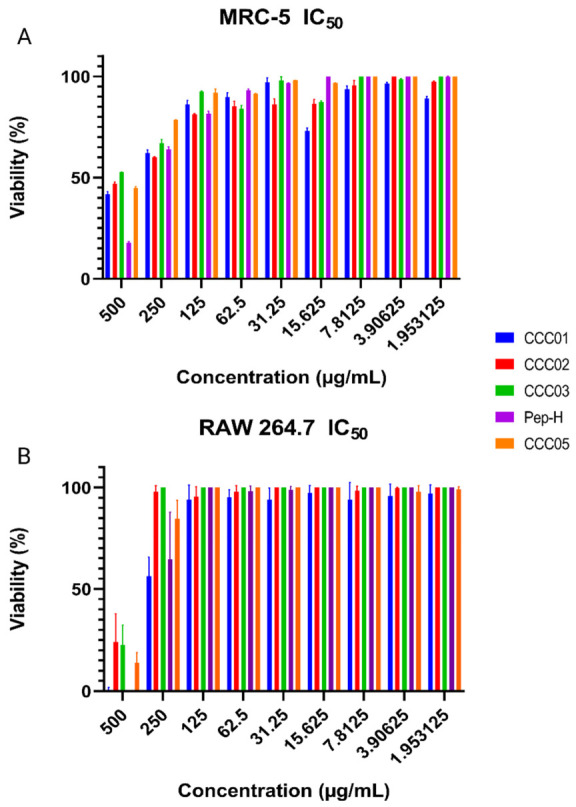
Cell viability of human lung fibroblasts MRC-5 and murine macrophages RAW 264.7 following exposure to the AMPs. Cell viability was evaluated over a concentration range of 1.95 to 500 μg mL^−1^. (**A**) Cell viability in MRC-5. (**B**) Cell viability in RAW 264.7. Data are expressed as mean ± SD from three independent experiments, *n* = 3.

The complete computationally prioritized peptide panel was evaluated in human lung fibroblasts MRC-5 and murine macrophages RAW 264.7 (Table 4). The IC_50_ values were used to determine whether the substitutions altered mammalian-cell compatibility and to calculate selectivity indices relative to the corresponding MIC_90_ values. In MRC-5, Pep-H, which retains Cys at the evaluated position, exhibited the highest relative cytotoxicity within the series (Figure 2A), with an IC_50_ value of 325.66 ± 5.46 μg mL^−1^. Substitution with Trp in CCC01 partially reduced this effect in MRC-5, increasing the IC_50_ to 399.64 ± 16.35 μg mL^−1^, whereas substitutions with Ala and Met resulted in values above 440 μg mL^−1^. Substitution with Arg in CCC03 displayed the most favorable profile in this cell lineage, as it did not reach 50% cellular inhibition within the evaluated concentration range.

In RAW 264.7, the pattern changed (Figure 2B). CCC01, which incorporates Trp, displayed an IC_50_ of 274.84 ± 37.07 μg mL^−1^, comparable to Pep-H, which showed an IC_50_ of 294.65 ± 68.70 μg mL^−1^. These results indicate that replacement of Cys by Trp maintained a cytotoxicity profile comparable to Pep-H in RAW 264.7 cells, whereas the two peptides differed in MRC-5 cells (CCC01: 399.64 ± 16.35 μg mL^−1^; Pep-H: 325.66 ± 5.46 μg mL^−1^). Despite this higher relative toxicity, Pep-H maintained the highest selectivity indices in both cell lineages due to its greater potency against *Mtb* H37Rv.

### 2.5. Hemolytic Activity

**Figure 3:**
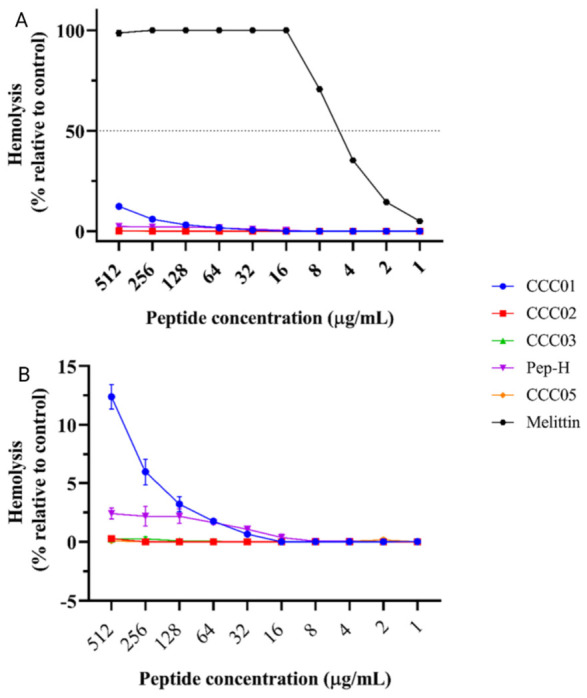
Hemolytic activity of the AMPs in erythrocytes. (**A**) Hemolytic profile of the AMPs compared with melittin. (**B**) Expanded view of the hemolytic profile of the AMPs excluding melittin. Data are expressed as mean ± SD from three independent experiments, *n* = 3.

The peptide series exhibited low hemolytic activity throughout the entire concentration range evaluated. None of the analogs reached 50% hemolysis at concentrations up to 512 μg mL^−1^, indicating that the HC_50_ values were above the highest concentration tested for CCC01, CCC02, CCC03, Pep-H, and CCC05 (Figure 3). Melittin displayed the expected profile of the hemolytic control, with nearly complete hemolysis from intermediate concentrations onward and a concentration-dependent response at lower concentrations. Among the tested peptides, CCC01 produced the highest, although still limited, hemolytic response, which became evident at the upper concentration range and remained below 13% even at 512 μg mL^−1^. This peptide, corresponding to the Cys-to-Trp substitution in the sequence RRYGTWIYQGRLWAF-NH_2_, reached 12.39 ± 1.46% hemolysis at 512 μg mL^−1^ and 5.97 ± 1.57% at 256 μg mL^−1^. Although these values remained well below the 50% threshold, CCC01 separated from the remaining analogs and exhibited significantly higher hemolysis at the upper concentrations according to statistical comparison. Within the evaluated panel, the Cys-to-Trp substitution was associated with the greatest hemolytic effect, consistent with an increased interaction of CCC01 with erythrocyte membranes.

Pep-H displayed low hemolytic activity despite retaining the native Cys residue of the parental motif. At 512 μg mL^−1^, Pep-H produced 2.41 ± 0.65% hemolysis, whereas at 256 μg mL^−1^ it reached 2.18 ± 1.19%, remaining at low levels even at concentrations substantially higher than its inhibitory concentration against *Mtb* H37Rv. CCC02, CCC03, and CCC05 exhibited even lower hemolytic profiles, with percentages close to basal levels throughout the evaluated range. At the highest concentration tested, CCC02, CCC03, and CCC05 displayed hemolysis values of 0.27 ± 0.32%, 0.25 ± 0.35%, and 0.10 ± 0.14%, respectively.

### 2.6. Ethidium Bromide Accumulation in Mtb H37Rv

**Figure 4:**
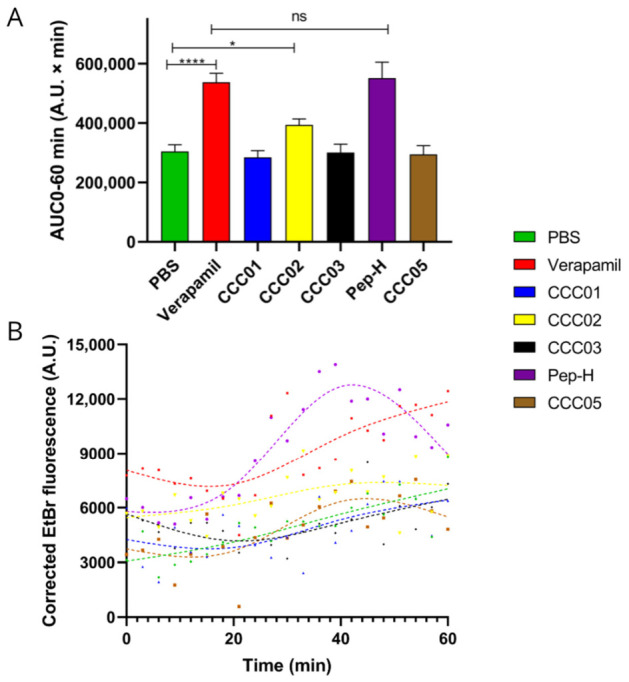
Ethidium bromide accumulation in *Mtb* H37Rv following exposure to the AMPs. Intracellular EtBr accumulation was monitored by fluorometry every 3 min for 60 min in the presence of the AMPs at ½ MIC. PBS was used as the untreated reference condition, and verapamil was used as an efflux-inhibition control. (**A**) Kinetic profiles of corrected EtBr fluorescence expressed in arbitrary units (A.U.). For each replicate and time point, the fluorescence of the corresponding cell-free background containing EtBr and the same treatment was subtracted from the bacteria-containing condition. Points represent the mean corrected fluorescence of three replicates. (**B**) Area under the corrected fluorescence–time curve from 0 to 60 min, calculated separately for each replicate using the trapezoidal method and expressed as A.U. × min. Bars represent mean ± SD, *n* = 3. Statistical comparisons were performed using the replicate AUC values. AUC, area under the curve; A.U., arbitrary units; ns, not significant; * *p* < 0.05; **** *p* < 0.0001.

The EtBr accumulation kinetics displayed distinct profiles among the evaluated AMPs (Figure 4A). The verapamil control produced the expected increase in corrected fluorescence relative to the basal condition, consistent with increased intracellular retention of the fluorophore. Pep-H exhibited a behavior distinct from the remaining analogs, characterized by a marked increase in signal throughout the assay and an approximate maximum around 40 min, followed by a partial decrease in fluorescence. Although the corrected AUC of Pep-H was comparable to that of verapamil, with no statistically significant difference between both treatments, the shape of the curve suggests a different accumulation kinetic profile (Figure 4B).

Substitution of Cys modified the EtBr accumulation profile. CCC01, CCC03, and CCC05 remained close to the basal control, whereas CCC02 displayed a partial increase without reaching the response observed for Pep-H or verapamil. The contrast between Pep-H and CCC05 is particularly informative because both contain sulfur-bearing residues, but only Pep-H retains the Cys thiol. The inability of CCC05 to reproduce the Pep-H profile suggests that sulfur retention alone is insufficient and that thiol chemistry, sulfur position, side-chain geometry, or their combined effects may contribute to the observed response.

### 2.7. Antioxidant Activity

**Figure 5:**
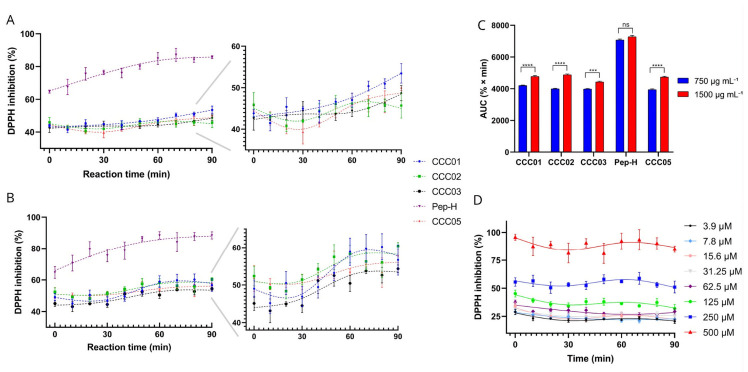
Antioxidant activity of the AMPs through DPPH radical inhibition. DPPH inhibition was monitored for 90 min for the AMPs at 750 and 1500 μg mL^−1^. (**A**) DPPH inhibition kinetics at 750 μg mL^−1^, with an enlarged view of the region containing the overlapping analog profiles. (**B**) DPPH inhibition kinetics at 1500 μg mL^−1^, with an enlarged view of the corresponding overlapping region. (**C**) Area under the curve of DPPH inhibition at both concentrations. (**D**) Inhibition kinetics of ascorbic acid used as the antioxidant control. Data are expressed as mean ± SD, *n* = 3. Statistical comparisons were performed between 750 and 1500 μg mL^−1^ for each AMP. ns, not significant; *** *p* < 0.001; **** *p* < 0.0001.

The antioxidant capacity of the AMPs was evaluated through DPPH inhibition at 750 and 1500 μg mL^−1^ for 90 min (Figure 5). Pep-H exhibited the highest profile within the series at both concentrations and displayed a time-dependent ascending kinetic behavior. At 750 μg mL^−1^, Pep-H started with 64.76 ± 0.93% inhibition and reached 85.72 ± 0.88% after 90 min. At 1500 μg mL^−1^, a similar pattern was observed, with 64.71 ± 3.73% at the initial time point and 88.40 ± 2.28% at the end of the assay. AUC analysis confirmed this behavior, with values of 7069.03 ± 20.47% at 750 μg mL^−1^ and 7277.05 ± 175.29% at 1500 μg mL^−1^, without statistically significant differences between both concentrations. These results indicate that Pep-H reached a high inhibition level from 750 μg mL^−1^ onward and that increasing peptide concentration did not significantly enhance the overall response.

The analogs generated through Cys substitution exhibited lower inhibition percentages and considerably flatter kinetics than Pep-H. At 750 μg mL^−1^, final DPPH inhibition values remained within an approximate range of 45–55%, whereas at 1500 μg mL^−1^ they increased partially to approximately 54–60%. Consistently, AUC values for these analogs ranged around 3900–4200% at 750 μg mL^−1^ and increased significantly to approximately 4400–4900% at 1500 μg mL^−1^. The weaker response of CCC05 indicates that retention of sulfur as a Met thioether was insufficient to reproduce the antioxidant behavior of the Cys-containing peptide.

Ascorbic acid displayed a kinetic profile distinct from that of Pep-H. At 500 μM, the antioxidant control showed high inhibition from the beginning of the assay, reaching 95.83 ± 2.67%, and remained elevated throughout the experiment, ending with 85.06 ± 2.74% inhibition at 90 min. In contrast, Pep-H started from lower values and progressively increased over time. This kinetic difference suggests that ascorbic acid acts through a rapid and sustained response from the earliest time points, whereas Pep-H develops a progressive DPPH neutralization throughout the reaction.

## 3. Discussion

The present study used Pep-H as a compact scaffold derived from HNP-1 to evaluate the contribution of a single Cys residue within the RRYGTXIYQGRLWAF motif. Because all analogs retained the same peptide length and surrounding sequence, the observed differences can be linked to replacement of residue X, enabling residue-level comparison within the Pep-H scaffold. Although computational models classified all selected analogs as antitubercular, the experimental results revealed a clear hierarchy that was not quantitatively predicted, and only Pep-H reached the experimental antimycobacterial activity threshold adopted in this study, defined as MIC_90_ < 25 μg mL^−1^ against *Mtb* H37Rv. This is consistent with the role of AMP prediction platforms as prioritization tools rather than substitutes for biological validation, since peptide activity depends on factors such as sequence context, membrane interaction, aggregation, permeability, stability, and compatibility with host cells [11,12,13].

The antimycobacterial assay identified Cys as the preferred residue among those tested at position X of Pep-H. Substitution of Cys reduced activity against *Mtb* H37Rv in all cases, supporting a contribution of the native residue to the antimycobacterial profile. CCC03 remained the closest analog despite increasing the estimated net charge from +3 to +4, indicating that enhanced cationicity alone was insufficient to preserve activity. The Cys-to-Arg replacement also introduced a longer guanidinium-containing side chain with different hydrogen-bonding, steric, and charge-distribution properties; experimental AMP studies show that the effect of Arg substitution depends on residue proportion and position within the complete sequence rather than on net charge alone [14]. CCC05 retained sulfur but did not reproduce the potency of Pep-H, supporting a possible contribution of Cys-specific chemistry. However, Met introduces a longer side chain and places sulfur farther from the peptide backbone, and model-peptide studies show that thioether proximity to the backbone can alter sulfur-centered reaction pathways [15]. The Pep-H–CCC05 difference therefore cannot be assigned exclusively to the replacement of a thiol by a thioether. CCC01 combined reduced potency with the highest hemolytic response, suggesting that the aromatic bulk and hydrophobicity introduced by Trp may have altered membrane interaction; experimental redesign of AMPs likewise shows that aromatic content, cationicity, topology, and amphipathic organization act jointly in determining activity and selectivity [16]. CCC02 produced the greatest potency loss, indicating that Ala was the least tolerated replacement at this position. Together, these findings support a privileged but multifactorial role for Cys within the GTCIY segment rather than an effect governed exclusively by a single electronic or structural property. Pep-H remained substantially less potent than rifampicin, and the present findings do not support its consideration as a direct alternative to the reference drug in its current form. Rifampicin was included as an established pharmacological reference for the susceptibility assay, whereas the peptide panel was evaluated to determine whether the computationally prioritized substitutions produced measurable antimycobacterial activity and an acceptable relationship between activity and mammalian-cell compatibility.

Cytotoxicity was evaluated as a complementary component of the comparative peptide characterization rather than as a subsequent lead-advancement decision based on equivalence to rifampicin. This assessment was necessary because MIC values alone cannot determine whether an amino-acid substitution produces a favorable activity–toxicity relationship. Although Pep-H was not the least cytotoxic peptide in absolute terms, it displayed the highest selectivity indices within the evaluated panel because it retained the greatest antimycobacterial activity. These results identify the most favorable activity–compatibility relationship among the peptides examined, while indicating that substantial potency optimization would be required before translational development could be considered [17,18]. The entire series displayed low hemolytic activity, with HC_50_ values above the maximum concentration tested. CCC01 nevertheless showed a modest increase in hemolysis at the highest concentrations. This pattern is consistent with altered erythrocyte-membrane interaction after the Cys-to-Trp substitution, although it cannot be attributed exclusively to the indole group because the replacement also changes side-chain dimensions, local hydrophobicity, and peptide organization. Experimental AMP-design studies similarly show that membrane activity and host–cell selectivity arise from the balance among charge, hydrophobicity, amphipathicity, and sequence organization [19]. Pep-H combined the highest antimycobacterial potency in the panel with low hemolysis, resulting in the most favorable activity–hemolysis balance among the tested peptides.

The EtBr accumulation assay revealed an additional functional difference between Pep-H and its analogs. Pep-H produced a marked increase in EtBr-associated fluorescence, with an AUC comparable to that of verapamil, although with a distinct kinetic profile. Because EtBr accumulation reflects the combined effects of fluorophore uptake, intracellular retention, permeability, and efflux, this result should not be interpreted as direct evidence of pump inhibition [20,21]. In mycobacteria, membrane depolarization can impair energy-dependent efflux and increase intracellular substrate retention [22]. The present data therefore support a Pep-H-associated increase in EtBr retention but do not distinguish direct efflux-pump inhibition from altered membrane permeability or cellular energetics. CCC01, CCC03, and CCC05 remained close to the basal profile, whereas CCC02 showed only a partial increase, indicating that this response was strongest in the Cys-containing peptide.

The antioxidant assay provided the strongest functional support for a contribution of Cys chemistry. Pep-H showed the highest DPPH inhibition and a progressive kinetic profile, consistent with the recognized influence of amino-acid composition and sulfur-containing residues on antioxidant peptide activity [23]. The comparison with CCC05 is particularly informative because Met retains sulfur as a thioether but differs from Cys in side-chain length, sulfur position, nucleophilicity, and local geometry. The weaker response of CCC05 therefore supports a contribution of thiol-specific chemistry, although it does not establish thiol loss as the sole determinant of the observed difference. Antioxidant peptide activity is also influenced by residue identity and position, peptide hydropathy, molecular weight, and the surrounding sequence environment [24]. Under the evaluated conditions, Met did not reproduce the antioxidant behavior of the Cys-containing peptide. The distinct profiles of Pep-H and ascorbic acid further indicate different reaction kinetics, and the time-dependent nature of DPPH responses should be considered when comparing antioxidant compounds [25].

The focused substitution design enabled direct comparison of chemically distinct residues at a conserved sequence position. However, each replacement simultaneously altered several interdependent properties, including side-chain dimensions, charge distribution, hydrophobicity, hydrogen-bonding capacity, sulfur position, acid–base behavior, nucleophilicity, and local conformational preferences. The observed biological differences therefore support residue-specific trends and chemically plausible mechanistic hypotheses, but they do not establish a unique molecular mechanism or independently quantify the contribution of each physicochemical variable. Broader substitution panels and experimentally constrained structural or membrane-interaction studies will be required to distinguish these contributions.

Taken together, the results identify Cys as the preferred residue among those tested within the GTCIY segment of Pep-H. Arg and Met produced the closest antimycobacterial activities, but neither increased cationicity nor sulfur retention was sufficient to reproduce the parental profile. Trp was associated with reduced potency and increased hemolysis, whereas Ala produced the greatest activity loss. These trends support a multifactorial contribution of Cys side-chain chemistry, sulfur position, and the surrounding structural environment rather than a mechanism governed exclusively by the thiol group. Future optimization should therefore retain Cys as an empirical design constraint while exploring flanking residues, terminal modifications, or other structural features capable of improving potency, stability, solubility, and selectivity.

## 4. Materials and Methods

### 4.1. Materials

Middlebrook 7H9 broth supplemented with oleic acid, albumin, dextrose, and catalase (OADC) was purchased from Difco Laboratories (Detroit, MI, USA). Dulbecco’s modified Eagle’s medium (DMEM) was obtained from Vitrocell (São Paulo, Brazil). Fetal bovine serum, gentamicin sulfate (BioReagent), amphotericin B (BioReagent), resazurin sodium salt, ethidium bromide (EtBr, 95%), verapamil hydrochloride, dimethyl sulfoxide (DMSO, 99.9%), hydroxybenzotriazole (HOBt, 98%), diisopropylcarbodiimide (DIC, 99%), trifluoroacetic acid (TFA, 99%), triisopropylsilane (TIS, 98%), 1,2-ethanedithiol (EDT, 98%), and Tween 80 (99%) were supplied by Merck KGaA (Darmstadt, Germany). Dimethylformamide (DMF, 99.8%) was purchased from Neon Comercial (São Paulo, Brazil), and dichloromethane (DCM, 99.8%) was purchased from Anidrol Produtos para Laboratórios (Diadema, São Paulo, Brazil). Phosphate-buffered saline (PBS) was freshly prepared in-house from analytical-grade reagents and ultrapure water. All amino acids used for peptide synthesis were purchased from AAPPTEC (Louisville, KY, USA).

### 4.2. Methods

#### 4.2.1. In Silico Assays

For the in silico analyses, an antimycobacterial peptide fragment derived from human neutrophils and previously reported for its biological potential was selected as the parental sequence. To evaluate the effect of amino acid substitutions on antimycobacterial activity, a systematic substitution of the cysteine residue present in the original sequence was performed by individually replacing it with each of the standard amino acids. The generated sequences were analyzed using the AntiTBPred platform (https://webs.iiitd.edu.in/raghava/antitbpred/ (accessed on 6 July 2025)) employing the four predictive methodologies available, MD-SVM, MD-hybrid, RD-SVM and RD-hybrid [26]. These approaches enabled estimation of the antimycobacterial potential of each peptide variant using computational models based on physicochemical properties and structural patterns associated with AMPs. Additionally, the hemolytic potential of the selected sequences was evaluated using the HemoPI2 platform (https://webs.iiitd.edu.in/raghava/hemopi2/ (accessed on 6 July 2025)) in order to predict possible cytotoxic effects on human erythrocytes and preliminarily assess biological safety [27]. Finally, peptide stability and half-life were estimated using the PlifePred server (http://webs.iiitd.edu.in//raghava/plifepred/ (accessed on 6 July 2025)), which predicts peptide stability under different biological conditions based on amino acid sequence-derived models [28].

#### 4.2.2. Peptide Synthesis and Cleavage

The AMPs were synthesized by SPPS using the Fmoc strategy. Initially, Fmoc-Rink amide resin was conditioned for 15 min in DMF prior to addition of the first amino acid. Deprotection of the Fmoc group was carried out using 20% 4-methylpiperidine in DMF. Sequential amino acid coupling reactions were performed using HOBt and DIC as coupling agents, maintaining the reaction mixture under stirring at room temperature for 2 h. Washing cycles were performed exclusively with DMF. After each coupling step, the Kaiser test was performed using 0.2% ninhydrin in ethanol to verify coupling efficiency, where the absence of blue coloration was interpreted as completion of the reaction. After completion of peptide assembly, the AMPs were cleaved and globally deprotected from the resin by acidolysis using a mixture of TFA, TIS, EDT, and water (94:2.5:2.5:1, *v*/*v*). The filtrate was concentrated under a nitrogen stream, and the peptide was precipitated dropwise in cold diethyl ether, followed by centrifugation and multiple washing steps with the same solvent. Finally, the products were lyophilized to obtain dry peptides and stored at −20 °C until further analysis [29].

#### 4.2.3. Characterization and Purification of the AMPs

Crude peptide samples were analyzed by reverse-phase high-performance liquid chromatography (RP-HPLC) using a Shimadzu Prominence system (Shimadzu Corporation, Kyoto, Japan) equipped with an Agilent C18 analytical column (4.6 × 250 mm, 5 μm; Agilent Technologies, Santa Clara, CA, USA). Analytical runs were performed using a linear gradient from 5% to 95% mobile phase B over 30 min at a flow rate of 1 mL min^−1^ to establish peptide retention times and evaluate crude sample profiles. Mobile phase A was water containing 0.045% trifluoroacetic acid (TFA), whereas mobile phase B was acetonitrile containing 0.036% TFA. Peptide purification was subsequently performed by semipreparative RP-HPLC using a Luna C18 column (250 × 10 mm, Phenomenex, Torrance, CA, USA) at a flow rate of 5 mL min^−1^. Individual purification gradients were established for each peptide according to their analytical retention times (Table 5). Fractions corresponding to the target peptides were collected, lyophilized, and stored at −20 °C until further use. Peptide molecular masses and identities were confirmed by liquid chromatography–mass spectrometry (LC–MS) using a Prominence/Amazon SL HPLC–MS system. Chromatographic purity was determined by integration of analytical RP-HPLC peaks monitored at 220 nm, and all purified peptides showed purity values greater than 98% [30].

#### 4.2.4. Resazurin Microtiter Assay (REMA)

The REMA was used to evaluate bacterial viability in the presence of antimicrobial compounds and to determine the minimum inhibitory concentration against *Mtb* H37Rv (ATCC 27294), following the protocol described by Campos et al. [31]. A bacterial suspension adjusted to 10^5^ CFU mL^−1^ was prepared, and 100 μL were dispensed into each well of a 96-well plate, except for wells reserved for medium and compound controls. Subsequently, 100 μL of the compounds under investigation were added and serial dilutions were performed directly in the plate. Microplates were incubated for 7 days at 37 °C under a 5% CO_2_ atmosphere. After incubation, 30 μL of 0.01% resazurin previously dissolved in sterile distilled water were added to each well. Resazurin acted as a bacterial viability indicator through its reduction to fluorescent resorufin in the presence of metabolically active cells. Following an additional 24 h incubation period, fluorescence was recorded using a Cytation 3 system (Biotek, Santa Clara, CA, USA).

#### 4.2.5. In Vitro Cytotoxicity Assays

To compare mammalian-cell compatibility across the computationally prioritized peptide panel, determine IC_50_ values, and calculate selectivity indices relative to antimycobacterial activity, in vitro cytotoxicity assays were performed following the methodology described by Singh et al. [32]. MRC-5 cells were selected as a human lung-derived fibroblast line for a general pulmonary host–cell compatibility screen [33], whereas RAW 264.7 cells were selected for a macrophage-based host–cell compatibility screen because macrophages constitute a major intracellular host compartment during *Mtb* infection [34]. These cell lines were used as complementary preliminary toxicity models and were not intended to reproduce the cellular complexity of human tuberculous lung lesions or infected primary human macrophages. RAW 264.7 and MRC-5 cells were cultured in DMEM supplemented with 10% fetal bovine serum, 50 mg L^−1^ gentamicin sulfate, and 2 mg L^−1^ amphotericin B. Cells were maintained in culture flasks at 37 °C in a humidified atmosphere containing 5% CO_2_ until complete confluence was reached. Subsequently, cell density was adjusted to 2.5 × 10^5^ cells mL^−1^, and 100 μL were distributed into each well of 96-well plates. After 24 h of incubation, cell adhesion was verified prior to treatment. The compounds were evaluated at concentrations ranging from 1.95 to 500 μg mL^−1^, and the plates were incubated for 24 h at 37 °C under a 5% CO_2_ atmosphere using media free of antimicrobial and antifungal agents. After incubation, 30 μL of 0.01% resazurin solution were added to each well. Following an additional 3 h incubation period, fluorescence was quantified using a Cytation 3 system (Biotek, Santa Clara, CA, USA).

#### 4.2.6. Hemolytic Activity Assay

The hemolytic activity of the peptides was evaluated following the methodology described by Santos-Filho et al. [35], with modifications. Initially, each peptide was dissolved in PBS (pH 7.4) to obtain stock solutions equivalent to 1 mg in 0.5 mL. Serial dilutions were prepared in PBS, resulting in concentrations ranging from 1 to 512 μg mL^−1^. All assays were performed in triplicate. In parallel, a 4% suspension of human erythrocytes in PBS was prepared. PBS was used as the negative control, corresponding to the absence of hemolysis, whereas 1% Triton X-100 was employed as the positive control for total hemolysis. Melittin was used as a hemolytic peptide control due to its well-established lytic activity against erythrocyte membranes. Subsequently, 100 μL of erythrocyte suspension were added to each peptide concentration, and the samples were incubated for 1 h at 37 °C. After incubation, samples were centrifuged at 500× *g* for 5 min, and the supernatants were transferred to microplates for spectrophotometric determination of absorbance at 540 nm using a microplate reader. The percentage of hemolysis was calculated using Equation (1):

(1) $$Hemolysis(\%):\;\frac{\left(sample\;absorbance-negative\;control\;absorbance\right)}{\left(positive\;control\;absorbance-negative\;control\;absorbance\right)}\;\times 100$$

#### 4.2.7. Ethidium Bromide Accumulation Assay

Ethidium bromide (EtBr) accumulation in *Mtb* H37Rv was evaluated by fluorometry as a functional approach to investigate the possible effect of the peptides on efflux systems. The bacterial inoculum was cultured in 7H9 medium supplemented with OADC at 37 °C until reaching an OD_600_ of 0.6–0.8. Subsequently, the suspension was centrifuged, washed with PBS pH 7.4 containing 0.05% Tween 80, and adjusted to an OD_600_ of 0.44. In a black 96-well microplate, 100 µL of bacterial suspension, 50 µL of EtBr at ½ MIC_90_, and 50 µL of the peptide under investigation or verapamil, used as an efflux inhibition control, also at ½ MIC_90_, were added. PBS, EtBr, bacterial suspension, bacteria + EtBr, bacteria + AMPs, and bacteria + EtBr + verapamil controls were included. The plate was incubated for 15 min at room temperature protected from light. Fluorescence was recorded every 3 min for 60 min at 37 °C using excitation/emission wavelengths of 530/25 nm and 590/20 nm, respectively. For each replicate and time point, the fluorescence measured in the corresponding cell-free well containing EtBr and the same treatment was subtracted from the fluorescence measured in the bacteria-containing well, according to Equation (2). For the untreated reference condition, corrected fluorescence was calculated by subtracting the fluorescence of the PBS + EtBr control from that of the bacteria + EtBr + PBS condition. For verapamil and each peptide, the corresponding PBS + EtBr + treatment condition was used as the cell-free background. Corrected fluorescence values were expressed in arbitrary units (A.U.) and plotted as a function of time. The area under the corrected fluorescence–time curve from 0 to 60 min was calculated separately for each replicate using the trapezoidal method according to Equation (3). Because corrected fluorescence was expressed in A.U. and time in minutes, the integrated AUC was expressed as A.U. × min. The three replicate AUC values were summarized as mean ± SD and used for statistical comparisons [36].

(2) $$F_{corr}\left(t_{i}\right)=F_{Bac+EtBr+Trt\;}\left(t_{i}\right)-F_{EtBr+Trt}(t_{i})$$

(3) $${AUC}_{0-60\min}=\sum_{i=0}^{19}\left[\frac{F_{corr}\left(t_{i}\right)+F_{corr}(t_{i+1})}{2}\right]\left(t_{i+1}-t_{i}\right)$$

where

$F_{corr}\left(t_{i}\right)$ = corrected fluorescence at time *tᵢ*.
$F_{Bac+EtBr+Trt\;}\left(t_{i}\right)$ = fluorescence measured in the bacteria-containing condition.
$F_{EtBr+Trt}(t_{i})$ = fluorescence measured in the corresponding cell-free background containing EtBr and the same treatment.
$t_{i}$ and $t_{i+1}$ = measurement time *i* and subsequent measurement time.
${AUC}_{0-60\min}$= area under the corrected fluorescence–time curve from 0 to 60 min, expressed as A.U. × min.

#### 4.2.8. DPPH Antioxidant Activity Assay

The antioxidant activity of the AMPs was evaluated using the DPPH radical scavenging method adapted from Hesamzadeh et al. [37]. Peptides CCC01–CCC05 and Pep-H were prepared at final concentrations of 1500 and 750 µg/mL. Each sample was mixed with a DPPH solution and incubated at room temperature protected from light. The reaction was monitored for 90 min, with periodic measurements recorded to determine the reduction in the DPPH signal. Ascorbic acid was used as a positive control at concentrations ranging from 3.9 to 500 µM. The percentage of DPPH radical inhibition was calculated by comparing the signal of each sample with that of the negative control according to Equation (4):

(4) $$DPPH\;radical\;scavenging\;activity\left(\%\right)=\;\frac{Absorbance\;of\;the\;negative\;control-Absorbance\;of\;the\;sample}{Absorbance\;of\;the\;negative\;control}$$

#### 4.2.9. Statistical Analysis

All experiments were performed in triplicate, and data are expressed as mean ± standard deviation. MIC_90_ values were determined from the REMA as the lowest peptide concentration producing at least 90% inhibition of bacterial viability relative to the untreated growth control. For comparative activity assessment, peptides with MIC_90_ values below 25 μg mL^−1^ were considered to meet the experimental antimycobacterial activity threshold adopted by our group. IC_50_ values for MRC-5 and RAW 264.7 cells were calculated by nonlinear regression using a four-parameter logistic dose–response model. Selectivity indices were calculated as the ratio between the IC_50_ value in each mammalian cell line and the MIC_90_ value against *Mtb* H37Rv. For hemolysis assays, the percentage of hemolysis was calculated relative to the negative control treated with PBS and the positive control treated with 1% Triton X-100. HC_50_ values were considered higher than the maximum tested concentration when 50% hemolysis was not reached. For the ethidium bromide accumulation assay, the AUC from 0 to 60 min was calculated separately for each replicate from the corrected fluorescence–time curve using the trapezoidal method. For the DPPH radical scavenging assay, kinetic curves were likewise analyzed by AUC calculation over the corresponding experimental period. Statistical comparisons were performed using one-way or two-way analysis of variance (ANOVA), followed by the appropriate multiple-comparison post hoc test. Differences were considered statistically significant when *p* < 0.05. Statistical significance was indicated as follows: ns, not significant; * *p* < 0.05; ** *p* < 0.01; *** *p* < 0.001; and **** *p* < 0.0001. Statistical analyses and graph generation were performed using GraphPad Prism 8 software.

#### 4.2.10. Use of Generative Artificial Intelligence

ChatGPT 5.5 (OpenAI) was used to generate an initial version of the graphical abstract based on scientific content and design instructions provided by the authors. The generated output was critically reviewed and manually revised to ensure consistency with the experimental results and interpretations presented in the manuscript. The tool was not used to generate experimental data, perform statistical analyses, select results, or formulate scientific conclusions. The complete prompt used for the graphical abstract is provided as Text S1 in the Supplementary Materials.

## 5. Conclusions

This study identified Cys as the preferred residue among the substitutions tested at position X of the RRYGTCIYQGRLWAF-NH_2_ motif. Replacement by Arg, Met, Trp, or Ala reduced antimycobacterial potency, and none of the analogs reproduced the combined selectivity, low hemolysis, EtBr accumulation, and antioxidant profile of Pep-H. Arg increased net positive charge and cellular tolerance but did not restore potency; Trp was associated with the highest hemolytic response and a cytotoxicity profile comparable to Pep-H in RAW 264.7 cells; Ala produced the greatest potency loss; and Met retained sulfur but did not reproduce the antimycobacterial, EtBr accumulation, or antioxidant behavior of the Cys-containing peptide. These outcomes support a multifactorial contribution of Cys side-chain chemistry and the local GTCIY environment rather than an exclusively thiol-dependent mechanism. Pep-H therefore remains the most suitable experimental reference for a subsequent optimization cycle focused on flanking residues, terminal modifications, proteolytic stability, solubility, and amphipathic balance, although substantial potency improvement will be required before translational development can be considered.

## Figures and Tables

**Table 1:** AMPs selected for the focused substitution panel through replacement of Cys within the RRYGTXIYQGRLWAF motif.

Peptide	Sequence	Cys Substitution	Key Physicochemical Features	Substituting Residue Structure
CCC01	RRYGT**W**IYQGRLWAF-NH_2_	Cys to Trp	Incorporation of an aromatic indole ring, increasing steric volume and local hydrophobicity.	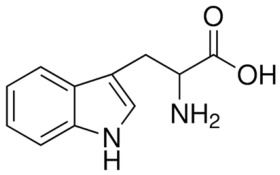
CCC02	RRYGT**A**IYQGRLWAF-NH_2_	Cys to Ala	Replacement by a small methyl side chain, reducing steric volume and functional complexity of the residue.	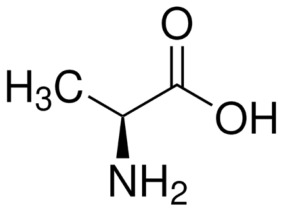
CCC03	RRYGT**R**IYQGRLWAF-NH_2_	Cys to Arg	Incorporation of a longer, flexible guanidinium-containing side chain with delocalized positive charge.	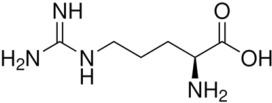
Pep-H	RRYGT**C**IYQGRLWAF-NH_2_	Conserved Cys	Retention of the thiol group, preserving a sulfur-containing residue with potential reactivity and polarizability.	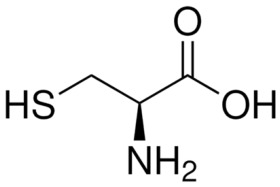
CCC05	RRYGT**M**IYQGRLWAF-NH_2_	Cys to Met	Replacement by a longer thioether-containing side chain, retaining sulfur at a more distal position while eliminating thiol functionality.	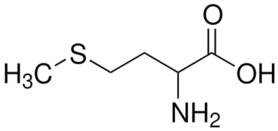

**Table 2:** Physicochemical properties and predicted half-life of the peptide analogs.

AMP	Half-Life (s)	Hydrophobicity	Hydropathicity	Hydrophilicity	Charge	Molecular Mass (Da)
CCC01	1147.21	−0.20	−0.67	−0.61	3.00	1973.49
CCC02	1197.51	−0.20	−0.49	−0.42	3.00	1858.35
CCC03	1015.91	−0.34	−0.91	−0.19	4.00	1943.46
Pep-H	1119.71	−0.22	−0.44	−0.45	3.00	1890.41
CCC05	1136.51	−0.20	−0.48	−0.47	3.00	1918.47

**Table 3:** Antimycobacterial activity of the AMPs against *Mtb* H37Rv.

AMP	MIC_90_ (μg mL^−1^)	Relative Change Compared to Pep-H
Pep-H	24.37 ± 0.07	1.00
CCC03	45.25 ± 1.01	1.86
CCC05	46.64 ± 0.54	1.91
CCC01	61.61 ± 7.16	2.53
CCC02	94.61 ± 2.19	3.88
Rifampicin	<0.098	-

**Table 4:** Cytotoxicity of the AMPs in human lung fibroblasts MRC-5 and murine macrophages RAW 264.7, and selectivity indices.

Sample	IC_50_ MRC-5 (μg mL^−1^)	IC_50_ RAW 264.7 (μg mL^−1^)	SI MRC-5	SI RAW 264.7
CCC01	399.64 ± 16.35	274.84 ± 37.07	6.49	4.46
CCC02	444.23 ± 12.01	416.27 ± 36.51	4.70	4.40
CCC03	>500	415.90 ± 20.04	>11.05	9.19
Pep-H	325.66 ± 5.46	294.65 ± 68.70	13.36	12.09
CCC05	462.50 ± 4.11	371.88 ± 7.41	9.92	7.97

**Table 5:** Retention times and purification gradients of the synthesized peptides obtained by semipreparative RP-HPLC. Peptides were purified by semipreparative reverse-phase high-performance liquid chromatography (RP-HPLC) using a Luna C18 column (250 × 10 mm) at a flow rate of 5 mL/min. The retention time and purification gradient of mobile phase B applied to each peptide are presented.

Peptide	Retention Time (min)	Linear Gradient
CCC01	16.2	25–70% mobile phase B over 120 min
CCC02	15.4	20–70% mobile phase B over 120 min
CCC03	14.9	15–70% mobile phase B over 120 min
Pep-H	15.5	20–70% mobile phase B over 120 min
CCC05	15.6	20–70% mobile phase B over 120 min

## Data Availability

All the relevant research data of this study can be found in the article or in the Supplementary Materials.

## Supplementary Materials

The following supporting information can be downloaded at: https://www.mdpi.com/article/10.3390/antibiotics15060618/s1. Table S1: Library of 20 peptide variants generated by cysteine permutation; Table S2: Prediction of peptide antitubercular activity using the MD-SVM model; Table S3: Prediction of peptide antitubercular activity using the MD-hybrid model; Table S4: Prediction of peptide antitubercular activity using the RD-SVM model; Table S5: Prediction of peptide antitubercular activity using the RD-hybrid model; Table S6: Hemolytic prediction of peptide analogs using HemoPI2; Figure S1: Chromatographic and mass spectrometric characterization of the synthesized peptide analogs; Text S1: Prompt used for the generation of the graphical abstract.
