# Supplementary material for: Comparative Biological and Functional Profiling of Single-Position Cysteine Substitutions in the HNP-1-Derived Peptide Pep-H Against Mycobacterium tuberculosis
Source: Antibiotics (Basel). 2026 Jun 17;15(6):618. doi: 10.3390/antibiotics15060618 (PMC13295688; doi:10.3390/antibiotics15060618)
Supplement: Supplementary file 1 [file antibiotics-15-00618-s001.zip › antibiotics-4362610-supplementary.pdf]

A single-position cysteine-centered structure–activity relationship study of Pep-H analogs against *Mycobacterium tuberculosis*

Christian S. Carnero Canales[1,*] Letícia Oliveira Catarin Nunes[2,3], Ariani Rodrigues Aragão[3], Norival Alves Santos Filho[2,3], Roxana Yesenia Pastrana Alta[1,*], Fernando Rogério Pavan[2,*]

[1] BIOMET, Laboratorio de Química Bioinorgánica en Medicina, Medioambiente y Tecnología, Facultad de Ciencias de la Universidad Nacional de Ingeniería, Av. Túpac Amaru 210, Rímac, Lima, Peru

[2]School of Pharmaceutical Sciences, São Paulo State University (UNESP), 14800-903 Araraquara, Brazil.

[3] Institute of Chemistry, São Paulo State University, 14800-060 Araraquara, Brazil.

***Addresses for correspondence:** fernando.pavan@unesp.br (Fernando R. Pavan), christian.carnero.c@uni.pe (Christian S. Carnero) and rpastranaa@uni.edu.pe (Roxana Y. P. Alta)

Table S1. Library of 20 peptide variants generated by cysteine permutation.

Amino acid	Resulting sequence
A (Ala)	RRYGTAIYQGRLWAF
R (Arg)	RRYGTRIYQGRLWAF
N (Asn)	RRYGTNIYQGRLWAF
D (Asp)	RRYGTDIYQGRLWAF
C (Cys)	RRYGT**C**IYQGRLWAF (original)
E (Glu)	RRYGTEIYQGRLWAF
Q (Gln)	RRYGTQIYQGRLWAF
G (Gly)	RRYGTGIYQGRLWAF
H (His)	RRYGTHIYQGRLWAF
I (Ile)	RRYGTIIYQGRLWAF
L (Leu)	RRYGTLIYQGRLWAF
K (Lys)	RRYGTK IYQGRLWAF
M (Met)	RRYGTMIYQGRLWAF
F (Phe)	RRYGTFIYQGRLWAF
P (Pro)	RRYG TPIYQGRLWAF
S (Ser)	RRYGTSIYQGRLWAF
T (Thr)	RRYGT TIYQGRLWAF
W (Trp)	RRYGTWIYQGRLWAF
Y (Tyr)	RRYGTYIYQGRLWAF
V (Val)	RRYGTVIYQGRLWAF

Table S2. Prediction of peptide antitubercular activity using the MD-SVM model.

Sequence	Score	Prediction	Steric hindrance	Amphipathicity	Net Hydrogen	pI
RRYGTAIYQGRLWAF	1.766675085	Anti-TB	0.63	0.57	1.2	10.91
RRYGTWIYQGRLWAF	1.698341535	Anti-TB	0.63	0.57	1.27	10.91
RRYGTMIYQGRLWAF	1.666633835	Anti-TB	0.65	0.57	1.2	10.91
RRYGTLIYQGRLWAF	1.579743435	Anti-TB	0.63	0.57	1.2	10.91

RRYGTYIYQGRLWAF	1.543757535	Anti-TB	0.64	0.57	1.27	10.28
RRYGTVIYQGRLWAF	1.525060835	Anti-TB	0.64	0.57	1.2	10.91
RRYGTCIYQGRLWAF	1.513085305	Anti-TB	0.6	0.54	1.12	10.06
RRYGTEIYQGRLWAF	1.511895235	Anti-TB	0.64	0.66	1.27	9.99
RRYGTFIYQGRLWAF	1.492915235	Anti-TB	0.64	0.57	1.2	10.91
RRYGTHIYQGRLWAF	1.456359085	Anti-TB	0.6	0.67	1.27	10.91

Table S3. Prediction of peptide antitubercular activity using the MD-hybrid model.

Sequence	Score	Prediction	Steric hindrance	Amphipathicity	Net Hydrogen	pI
RRYGTAIYQGRLWAF	2.4356347	Anti-TB	0.63	0.57	1.2	10.91
RRYGTMIYQGRLWAF	2.3030142	Anti-TB	0.65	0.57	1.2	10.91
RRYGTLIYQGRLWAF	2.2686295	Anti-TB	0.63	0.57	1.2	10.91
RRYGTWIYQGRLWAF	2.2326907	Anti-TB	0.63	0.57	1.27	10.91
RRYGTHIYQGRLWAF	2.2016625	Anti-TB	0.60	0.67	1.27	10.91
RRYGTVIYQGRLWAF	2.1686740	Anti-TB	0.64	0.57	1.2	10.91
RRYGTCIYQGRLWAF	2.1187335	Anti-TB	0.60	0.54	1.12	10.06
RRYGTQIYQGRLWAF	2.0664193	Anti-TB	0.64	0.66	1.33	10.91
RRYGTIIYQGRLWAF	2.0641545	Anti-TB	0.64	0.57	1.2	10.91
RRYGTGIYQGRLWAF	2.0607799	Anti-TB	0.64	0.57	1.2	10.91

Table S4. Prediction of peptide antitubercular activity using the RD-SVM model.

Sequence	Score	Prediction	Steric hindrance	Amphipathicity	Net Hydrogen	pI
RRYGTRIYQGRLWAF	0.61888315	Anti-TB	0.64	0.74	1.47	11.56
RRYGTKIYQGRLWAF	0.57738517	Anti-TB	0.60	0.77	1.25	11.01

RRYGTWIYQGRLWAF	0.559664095	Anti-TB	0.63	0.57	1.27	10.91
RRYGTLIYQGRLWAF	0.542759375	Anti-TB	0.63	0.57	1.20	10.91
RRYGTHIYQGRLWAF	0.52113931	Anti-TB	0.60	0.67	1.27	10.91
RRYGTGIYQGRLWAF	0.51887171	Anti-TB	0.64	0.57	1.20	10.91
RRYGTYIYQGRLWAF	0.51263105	Anti-TB	0.64	0.57	1.27	10.28
RRYGTMIYQGRLWAF	0.47864097	Anti-TB	0.65	0.57	1.20	10.91
RRYGTIIYQGRLWAF	0.468194825	Anti-TB	0.64	0.57	1.20	10.91
RRYGTNIYQGRLWAF	0.461156995	Anti-TB	0.65	0.57	1.33	10.91

Table S5. Prediction of peptide antitubercular activity using the RD-hybrid model.

Sequence	Score	Prediction	Steric hindrance	Amphipathicity	Net Hydrogen	pI
RRYGTWIYQGRLWAF	2.3114519	Anti-TB	0.63	0.57	1.27	10.91
RRYGTKIYQGRLWAF	2.2809966	Anti-TB	0.60	0.77	1.25	11.01
RRYGTCIYQGRLWAF	2.2561526	Anti-TB	0.60	0.54	1.12	10.06
RRYGTVIYQGRLWAF	2.1608036	Anti-TB	0.64	0.57	1.20	10.91
RRYGTQIYQGRLWAF	2.1513460	Anti-TB	0.64	0.66	1.33	10.91
RRYGTYIYQGRLWAF	2.1175502	Anti-TB	0.64	0.57	1.27	10.28
RRYGTHIYQGRLWAF	2.0998991	Anti-TB	0.60	0.67	1.27	10.91
RRYGTPIYQGRLWAF	2.0895533	Anti-TB	0.62	0.57	1.20	10.91
RRYGTAIYQGRLWAF	2.0835720	Anti-TB	0.63	0.57	1.20	10.91
RRYGTFIYQGRLWAF	2.0731795	Anti-TB	0.64	0.57	1.20	10.91

Table S6. Hemolytic prediction of peptide analogs using HemoPI2.

AMPs	ESM Score	MERCI Score	Hybrid Score	Prediction
**CCC01**	0.344	-1.0	0.0	Non-Hemolytic
**CCC02**	0.272	-1.0	0.0	Non-Hemolytic
**CCC03**	0.235	-1.0	0.0	Non-Hemolytic
**Pep-H**	0.288	-1.0	0.0	Non-Hemolytic
**CCC05**	0.324	-1.0	0.0	Non-Hemolytic

*ESM: Evolutionary Scale Modeling; MERCI: Motif-EmeRging and with Classes-Identification

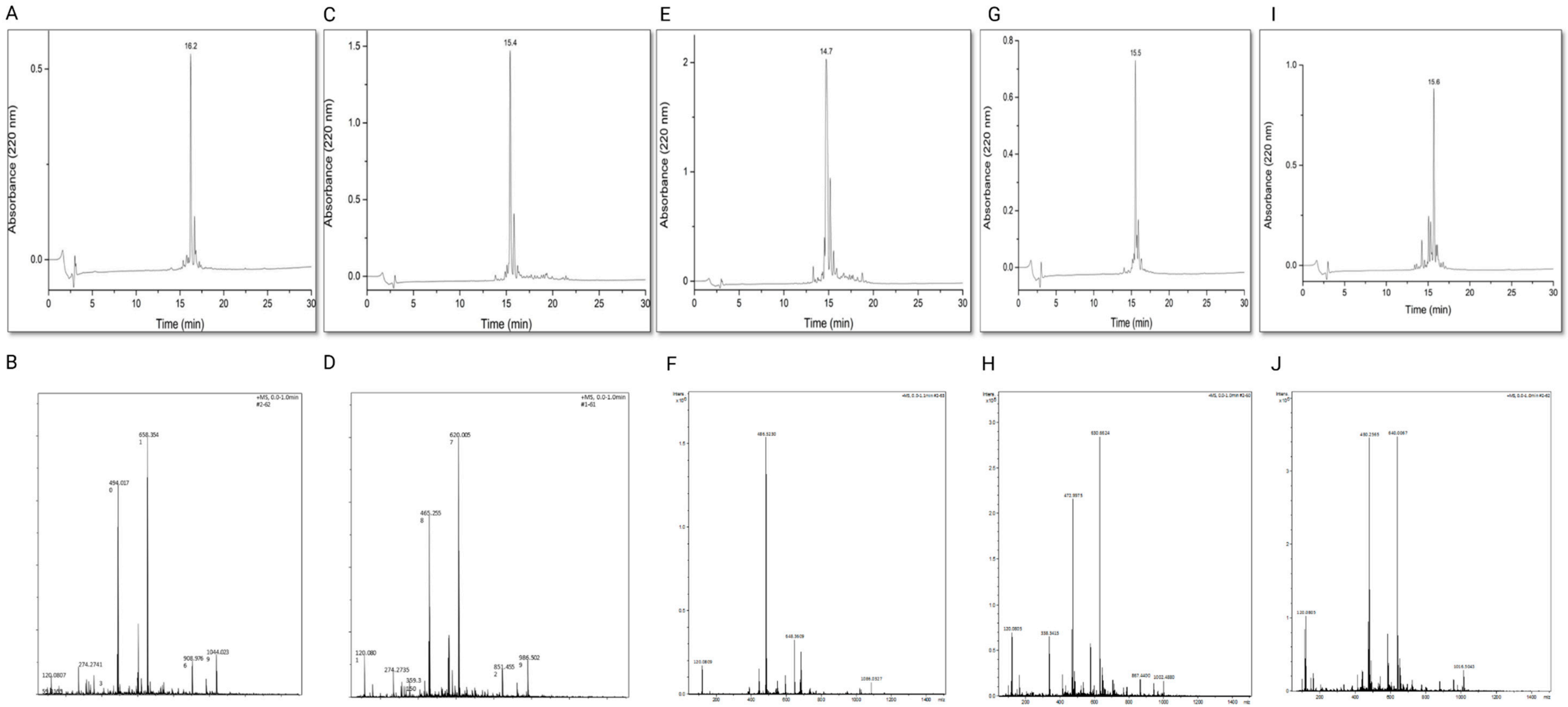

Figure S1. Chromatographic and mass spectrometric characterization of the synthesized peptide analogs. Panels A, C, E, G, and I show the analytical chromatograms recorded at 220 nm, while panels B, D, F, H, and J present the corresponding positive-mode mass spectra. Panels A–B correspond to CCC01, C–D to CCC02, E–F to CCC03, G–H to Pep-H, and I–J to CCC05, respectively.

Text S1. Prompt used for the generation of the graphical abstract

Create a high resolution scientific graphical abstract in a clean biomedical vector and 3D illustration style, suitable for a peer reviewed journal. The figure should summarize a cysteine centered structure activity relationship study of antimicrobial peptides derived from the HNP 1 motif against *Mycobacterium tuberculosis*.

Use a wide horizontal canvas with a white background, soft shadows, rounded rectangular panels, and a polished scientific design. Organize the composition around a central peptide scaffold labeled Pep H. In the upper center, show a short peptide chain made of connected glossy spheres. Use blue spheres for positively charged residues and gray or white spheres for neutral residues. Highlight the cysteine residue as a larger orange or gold sphere labeled C, with a visible thiol side chain labeled SH. Below the peptide, write Cys SH in orange gold text. Add blue positive charge signs around the peptide to indicate its cationic antimicrobial character. Add a soft yellow glow behind the cysteine thiol group to emphasize its functional importance.

In the upper left panel, titled Cys SAR, show four horizontal rows representing single position substitutions of the cysteine residue within the RRYGTXIYQGRLWAF NH2 motif. Each row should include a simplified peptide chain with the substituted residue highlighted inside a dotted circle. Represent Trp with a blue purple indole ring, Ala with a small blue methyl like sphere, Arg with a guanidinium side chain and positive charge signs, and Met with a green sulfur containing thioether side chain labeled S. At the bottom of this panel, include the text reduced anti Mtb activity to show that replacing cysteine decreases antimycobacterial activity.

In the upper right panel, titled Mtb, show two red rod shaped Mycobacterium tuberculosis bacilli with textured surfaces. Below them, draw a stylized mycobacterial cell envelope with curved multilayers, orange lipid chains, gray polar heads, and reddish inner layers. Place the Pep H peptide with the highlighted orange Cys SH group close to the bacterial membrane, suggesting interaction with the cell envelope. Add purple ethidium bromide like fluorescent spheres accumulating inside the bacterial cell. Include a circular inset with several purple spheres and the label increased EtBr accumulation.

In the lower part of the figure, create three outcome panels connected visually to the central Pep H peptide. In the left panel, titled low hemolysis, show intact glossy red blood cells with a protective shield and a check mark, indicating low hemolytic activity. In the center panel, titled increased selectivity, show a balance scale with a red Mtb bacillus on one side and a protected red blood cell on the other side, representing favorable selectivity toward Mtb over mammalian cells. In the right panel, titled reduced DPPH radical signal, show a purple DPPH radical changing into a green neutralized form, with a small Pep H fragment containing the orange Cys SH group above the reaction.

At the bottom of the figure, add a horizontal methods strip divided into six simple sections. Include icons labeled HPLC with blue chromatogram peaks, MS with a black mass spectrum, MIC with a blue microplate, EtBr with a dark microplate containing purple fluorescent wells, DPPH with a purple plate, and RBC with red blood cells.

The scientific message must be clear. Pep H is the lead analog because it preserves the Cys SH residue, which acts as a key chemical and positional determinant of biological activity. The cysteine substitutions by Trp, Ala, Arg, and Met define the SAR, but preservation of cysteine in Pep H supports the best overall profile, including stronger anti Mtb activity, increased EtBr accumulation, stronger DPPH antioxidant response, low hemolysis, and favorable selectivity.

Use crisp edges, readable text, high contrast colors, glossy molecular spheres, simplified but realistic biological structures, and a publication quality scientific aesthetic. Avoid overcrowding and do not include unnecessary decorative elements.
